# 123. Oral Ibrexafungerp Outcomes by Fungal Disease in Patients from an Interim Analysis of a Phase 3 Open-label Study (FURI)

**DOI:** 10.1093/ofid/ofab466.123

**Published:** 2021-12-04

**Authors:** Peter G Pappas, Oliver Cornely, Philipp Koehler, Todd P McCarty, Barbara D Alexander, Rachel Miller, Jose A Vazquez, John W Sanders, Caryn Morse, Luis Ostrosky-Zeichner, Robert Krause, Jürgen Prattes, Andrej Spec, Riina Rautemaa-Richardson, Rohit Bazaz, Thomas J Walsh, Francisco M Marty, Isabel H Gonzalez-Bocco, Marisa Miceli, Martin Hoenigl, Martin Hoenigl, Thomas F Patterson, Nkechi Azie, David A Angulo

**Affiliations:** 1 University of Alabama at Birmingham, Birmingham, Alabama; 2 University of Cologne, Faculty of Medicine and University Hospital Cologne, Cologne, Nordrhein-Westfalen, Germany; 3 University Hospital of Cologne, Cologne, Nordrhein-Westfalen, Germany; 4 University of Alabama at Birmingham; Birmingham VA Medical Center, Birmingham, Alabama; 5 Duke University, Durham, North Carolina; 6 Medical College of Georgia at Augusta University, Augusta, Georgia; 7 Wake Forest School of Medicine, Winston-Salem, North Carolina; 8 Wake Forest Baptist Hospital, Winston-Salem, North Carolina; 9 University of Texas Health Science Center, Houston, Houston, Texas; 10 Medical University of Graz, Section of Infectious Diseases and Tropical Medicine, Department of Internal Medicine, Graz, Steiermark, Austria; 11 Medical University of Graz, Graz, Steiermark, Austria; 12 Division of Infectious Diseases Washington University in St. Louis, ST LOUIS, MO; 13 University of Manchester, Manchester, England, United Kingdom; 14 Manchester University NHS Foundation Trust, Manchester, England, United Kingdom; 15 Weill Cornell Medicine, New York, NY; 16 Brigham and Women's Hospital, Boston, MA; 17 University of Michigan, Ann Arbor, Michigan; 18 UC San Diego, San Diego, California; 19 University of Texas Health San Antonio, San Antonio, TX; 20 SCYNEXIS, Inc., Jersey City, New Jersey

## Abstract

**Background:**

*Candida* species are a major cause of invasive and mucocutaneouls infections. There are limited oral treatment options available for patients with *Candida* infections who are unresponsive to or who are intolerant of currently available antifungals. Oral ibrexafungerp is an investigational broad-spectrum glucan synthase inhibitor antifungal with activity against *Candida* and *Aspergillus* species, including azole- and echinocandin-resistant strains. A Phase 3 open-label, single-arm study of ibrexafungerp (FURI; NCT03059992) is ongoing for the treatment of patients intolerant of or with fungal disease refractory to standard antifungal therapy. We present an analysis of patient outcomes from the FURI study by fungal disease type.

Table 1: FURI Outcomes by Fungal Disease

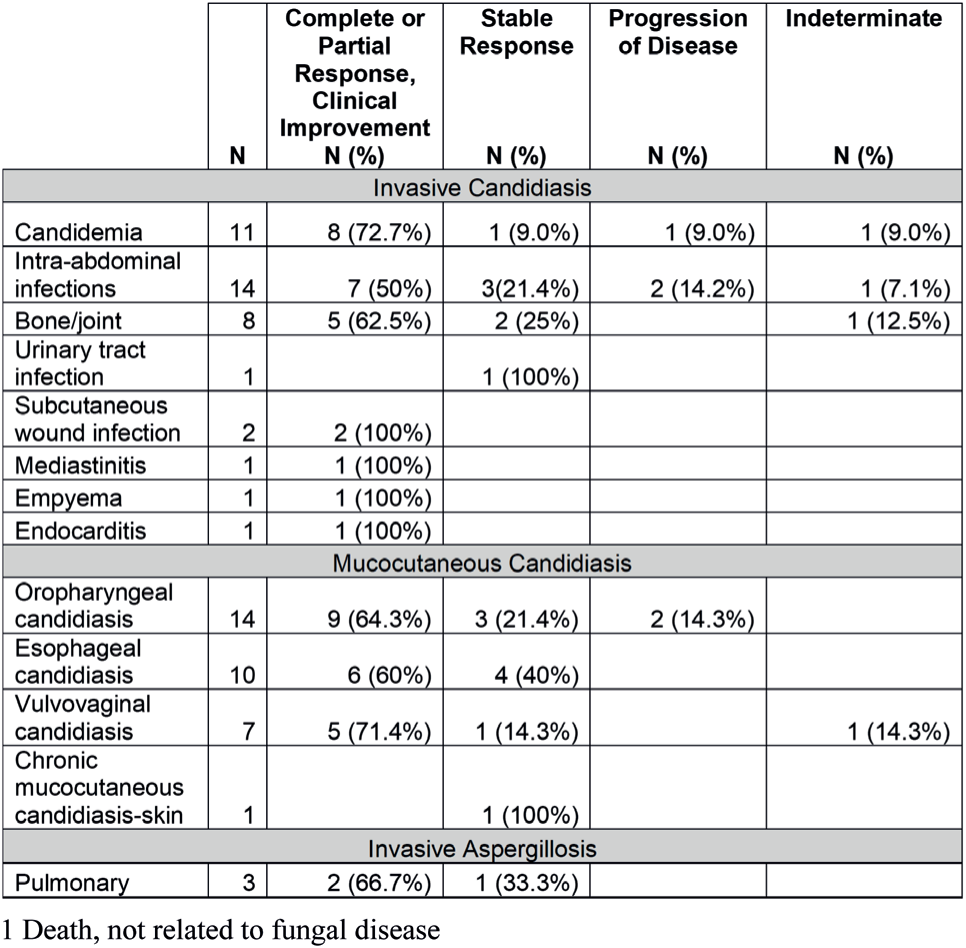

**Methods:**

FURI patients were eligible for enrollment if they have proven or probable, severe mucocutaneous candidiasis, invasive candidiasis or invasive aspergillosis,other fungal diseases and evidence of failure to, intolerance to, or toxicity related to a currently approved standard-of-care antifungal treatment or can not receive approved oral antifungal options (e.g., susceptibility of the organism) and a continued IV antifungal therapy is clinically undesirable or unfeasible.

**Results:**

An independent Data Review Committee (DRC) provided an assessment of treatment response for 74 patients enrolled in the FURI study from 22 centers in US, UK and EU treated with ibrexafungerp for mucocutaneous or invasive fungal infections from 2016- 2020. A total of 39 (52.7%) patients had invasive candidiasis, 32 (43.2%) had mucocutaneous candidiasis and 3 (4.5%) patients had invasive aspergillosis. The percent of patients who were determined to have a complete response (CR), partial response (PR), clinical improvement (CI) was 63.5%, stable disease (SD) was 23.0%, patients with progression of disease 6.8% and 4 patients were indeterminate. Additionally, there was 1 death in the FURI study that was not related to fungal disease. Table 1 shows outcomes by fungal disease type as determined by the DRC.

**Conclusion:**

Analysis of 74 patients from the FURI study indicates that oral ibrexafungerp provides a favorable therapeutic response in patients with challenging fungal disease and limited treatment options.

**Disclosures:**

**Peter G. Pappas, MD**, **Astellas** (Research Grant or Support)**Cidara** (Research Grant or Support)**F2G** (Consultant)**Matinas** (Consultant, Scientific Research Study Investigator)**Mayne Pharma** (Research Grant or Support)**Scynexis** (Research Grant or Support) **Oliver Cornely, Prof.**, **Actelion** (Consultant, Grant/Research Support)**Al-Jazeera Pharmaceuticals** (Consultant)**Allecra Therapeutics** (Consultant)**Amplyx** (Consultant, Grant/Research Support)**Astellas** (Consultant, Grant/Research Support)**Basilea** (Consultant, Grant/Research Support)**Biocon** (Consultant)**Biosys** (Consultant)**Cidara** (Consultant, Grant/Research Support)**CoRe Consulting** (Consultant)**Da Volterra** (Consultant, Grant/Research Support)**DFG (German Research Foundation**) (Grant/Research Support)**Entasis** (Consultant)**F2G** (Consultant, Grant/Research Support)**German Federal Ministry of Research and Education** (Grant/Research Support)**Gilead** (Consultant, Grant/Research Support)**Grupo Biotoscana** (Consultant)**Immunic** (Grant/Research Support)**IQVIA** (Consultant)**Janssen** (Grant/Research Support)**Matinas** (Consultant)**Medicines Company** (Grant/Research Support)**MedPace** (Consultant, Grant/Research Support)**Melinta Therapeutics** (Grant/Research Support)**Menarini** (Consultant)**Merck/MSD** (Consultant, Grant/Research Support)**Molecular Partners** (Consultant)**MSG-ERC** (Consultant)**Mylan** (Consultant)**Nabriva** (Consultant)**Noxxon** (Consultant)**Octapharma** (Consultant)**Paratek** (Consultant)**Pfizer** (Consultant, Grant/Research Support)**PSI** (Consultant)**Roche Diagnostics** (Consultant)**Scynexis** (Consultant, Grant/Research Support)**Seres** (Consultant)**Shionogi** (Consultant)**Wiley (Blackwell**) (Other Financial or Material Support) **Philipp Koehler, MD**, **Ambu GmbH** (Consultant, Speaker's Bureau)**Astellas Pharma** (Speaker's Bureau)**Euopean Confederation of Medical Mycology** (Speaker's Bureau)**German Federal Ministry of Research and Education** (Grant/Research Support)**Gilead** (Consultant, Speaker's Bureau)**MSD** (Speaker's Bureau)**Noxxon N.V.** (Consultant)**Pfizer** (Speaker's Bureau)**State of North Rhine-Westphalia, Germany** (Grant/Research Support) **Todd P. McCarty, MD**, **Cidara** (Grant/Research Support)**GenMark** (Grant/Research Support, Other Financial or Material Support, Honoraria for Research Presentation)**T2 Biosystems** (Consultant) **Barbara D. Alexander, MD, MHS**, **SCYNEXIS, Inc.** (Consultant) **Rachel Miller, MD**, **SCYNEXIS, Inc.** (Scientific Research Study Investigator) **Caryn Morse, MD**, **Chimerix** (Scientific Research Study Investigator)**Covis Pharma** (Scientific Research Study Investigator)**Gilead Sciences Inc.** (Scientific Research Study Investigator)**Ridgeback Biotherapeutics** (Scientific Research Study Investigator)**Roche** (Scientific Research Study Investigator)**SCYNEXIS, Inc.** (Scientific Research Study Investigator)**Theratechnologies** (Advisor or Review Panel member)**Viiv** (Advisor or Review Panel member) **Luis Ostrosky-Zeichner, MD**, **Amplyx** (Consultant)**Cidara** (Consultant)**F2G** (Consultant)**Gilead** (Grant/Research Support, Speaker's Bureau)**Pfizer** (Scientific Research Study Investigator, Speaker's Bureau)**Scynexis** (Grant/Research Support, Scientific Research Study Investigator)**Viracor** (Consultant) **Jürgen Prattes, Dr**, **AbbVie Inc.** (Shareholder)**Gilead** (Speaker's Bureau)**MSD** (Grant/Research Support)**Novo Nordisk** (Shareholder)**Pfizer** (Advisor or Review Panel member)**Stryker** (Shareholder) **Andrej Spec, MD, MSCI**, **Mayne Pharma** (Grant/Research Support) **Riina Rautemaa-Richardson, DDS, PhD, FRCPath**, **SCYNEXIS, Inc.** (Scientific Research Study Investigator) **Thomas J. Walsh, MD, PhD (hon**), **Scynexis** (Consultant, Grant/Research Support)**Shionogi** (Consultant, Grant/Research Support) **Francisco M. Marty, MD**, **SCYNEXIS, Inc.** (Scientific Research Study Investigator) **Marisa Miceli, MD**, **SCYNEXIS, Inc.** (Advisor or Review Panel member) **Martin Hoenigl, MD**, **Astellas** (Grant/Research Support)**Gilead** (Grant/Research Support)**Pfizer** (Grant/Research Support) **Martin Hoenigl, MD**, Astellas (Individual(s) Involved: Self): Grant/Research Support; F2G (Individual(s) Involved: Self): Grant/Research Support; Gilead (Individual(s) Involved: Self): Grant/Research Support; Pfiyer (Individual(s) Involved: Self): Grant/Research Support; Scýnexis (Individual(s) Involved: Self): Grant/Research Support **Thomas F. Patterson, MD**, **SCYNEXIS, Inc.** (Advisor or Review Panel member) **Nkechi Azie, MD**, **SCYNEXIS, Inc.** (Employee, Shareholder) **David A. Angulo, MD**, **SCYNEXIS, Inc.** (Employee, Shareholder)

